# Assessing the Role of Inhibition in Stabilizing Neocortical Networks Requires Large-Scale Perturbation of the Inhibitory Population

**DOI:** 10.1523/JNEUROSCI.0963-17.2017

**Published:** 2017-12-06

**Authors:** Sadra Sadeh, R. Angus Silver, Thomas D. Mrsic-Flogel, Dylan Richard Muir

**Affiliations:** ^1^Department of Neuroscience, Physiology, and Pharmacology, University College London, WC1E 6BT London, United Kingdom, and; ^2^Biozentrum, University of Basel, 4056 Basel, Switzerland

**Keywords:** computational model, cortical computation, inhibitory stabilization, optogenetics, recurrent excitation

## Abstract

Neurons within cortical microcircuits are interconnected with recurrent excitatory synaptic connections that are thought to amplify signals (Douglas and Martin, 2007), form selective subnetworks (Ko et al., 2011), and aid feature discrimination. Strong inhibition (Haider et al., 2013) counterbalances excitation, enabling sensory features to be sharpened and represented by sparse codes (Willmore et al., 2011). This balance between excitation and inhibition makes it difficult to assess the strength, or gain, of recurrent excitatory connections within cortical networks, which is key to understanding their operational regime and the computations that they perform. Networks that combine an unstable high-gain excitatory population with stabilizing inhibitory feedback are known as inhibition-stabilized networks (ISNs) (Tsodyks et al., 1997). Theoretical studies using reduced network models predict that ISNs produce paradoxical responses to perturbation, but experimental perturbations failed to find evidence for ISNs in cortex (Atallah et al., 2012). Here, we reexamined this question by investigating how cortical network models consisting of many neurons behave after perturbations and found that results obtained from reduced network models fail to predict responses to perturbations in more realistic networks. Our models predict that a large proportion of the inhibitory network must be perturbed to reliably detect an ISN regime robustly in cortex. We propose that wide-field optogenetic suppression of inhibition under promoters targeting a large fraction of inhibitory neurons may provide a perturbation of sufficient strength to reveal the operating regime of cortex. Our results suggest that detailed computational models of optogenetic perturbations are necessary to interpret the results of experimental paradigms.

**SIGNIFICANCE STATEMENT** Many useful computational mechanisms proposed for cortex require local excitatory recurrence to be very strong, such that local inhibitory feedback is necessary to avoid epileptiform runaway activity (an “inhibition-stabilized network” or “ISN” regime). However, recent experimental results suggest that this regime may not exist in cortex. We simulated activity perturbations in cortical networks of increasing realism and found that, to detect ISN-like properties in cortex, large proportions of the inhibitory population must be perturbed. Current experimental methods for inhibitory perturbation are unlikely to satisfy this requirement, implying that existing experimental observations are inconclusive about the computational regime of cortex. Our results suggest that new experimental designs targeting a majority of inhibitory neurons may be able to resolve this question.

## Introduction

Inspired by experimental observations of a repeated, “canonical” architecture for cortex (Creutzfeldt, 1977; Rockel et al., 1980; Muir et al., 2011), several groups of investigators have proposed that a concomitant canonical function might also exist, comprising a fundamental computational basis common to all cortical areas (Szentágothai, 1978; Douglas et al., 1989). How can this computational principle be discovered? A frequently applied approach in reverse engineering a complex dynamical system is to measure the response of a system to a perturbing stimulus. This technique has been applied to cortex in the past (Douglas et al., 1989), but recent methodological advances permit targeted stimulation or suppression of chosen neuronal populations through genetic targeting of light-sensitive ion channels and pumps: optogenetics (Boyden et al., 2005; Han and Boyden, 2007; Zhang et al., 2007; Atallah et al., 2012). Optogenetic stimulation can be used to drive or suppress the activity of genetically defined cell classes or cortical populations with particular projection targets. This approach confers the possibility of using carefully targeted perturbations to observe and detect the computational mode of cortex. However, due to the prevalence of recurrent interactions in cortical networks, the outcome of such a perturbation may be unintuitive or difficult to predict. For this reason, computational modeling of perturbations is required to relate network architectures and operating regimes to the expected result of a particular perturbation and to guide the choice of an appropriate experimental perturbation to test hypotheses optimally. Here, we take as a specific example the question of quantifying the excitatory/inhibitory balance in cortex, with a particular focus on mouse visual cortex.

Network computational mechanisms that rely on recurrent processing of information within cortex can be flexible and powerful (Hopfield, 1982; Douglas and Martin, 2007; Hopfield, 2015). Many computational models for mammalian cortex require strong recurrent excitation, which therefore must be balanced by strong local inhibition to maintain stability of the cortical network (Hahnloser, 1998; Rutishauser and Douglas, 2009; Neftci et al., 2013; Muir and Cook, 2014). Networks with this property are known as inhibition-stabilized networks (ISNs) (Tsodyks et al., 1997; Ozeki et al., 2009; Litwin-Kumar et al., 2016). An alternative configuration of cortical networks could rely on a weak excitatory population that is intrinsically stable, which would support different computational mechanisms not relying on strong excitatory recurrence. The question of which balanced regime mammalian neocortex operates in is therefore of experimental interest because this constrains the type of computations that could be supported by cortex. Anatomical and physiological estimates suggest that recurrent excitation is very strong, especially in the superficial layers of cortex (Binzegger et al., 2004; Lefort et al., 2009). Similarly, observations of epileptiform activity when inhibition is blocked in cortex suggest that inhibitory feedback is required for stability of the cortical network (Avoli et al., 1995; Mann et al., 2009). However, an ISN regime may also be detected functionally by perturbing the dynamics of cortical activity experimentally and observing the response of the network.

Here, we analyze theoretical and simulation models of cortical networks to determine the conditions under which an inhibitory perturbation evokes a measurable paradoxical response in the network, which can be used to infer the computational regime of cortex (Tsodyks et al., 1997). We then examine whether existing methods for perturbation of cortical activity such as electrical stimulation by injecting currents into inhibitory neurons, perfusion of the brain with chemical agonists or antagonists of inhibitory synaptic receptors (Bowery et al., 1984), or optogenetics will be able to reveal evidence for an ISN regime in cortex.

## Materials and Methods

### 

#### Neuron and network dynamics

We begin by defining a simple model for a cortical network containing equal numbers of excitatory and inhibitory linear threshold neurons (Wilson and Cowan, 1973). The activity dynamics of the network evolve according to the system of equations as follows:

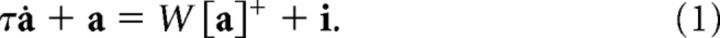
 Where τ is the activation time constant applied to all neurons in the network; **a** = (*x*_1_, *x*_2_, …, *x_N_*, *y*_1_, *y*_2_, …, *y_N_*)*^T^* is the vector of instantaneous activations (i.e., total input current in amps) of excitatory neurons *x*_i_ and inhibitory neurons *y*_i_ at time *t*; **ȧ** = d**a**/d*t*; **i** = (ι_1_, ι_2_, …, ι_2*N*_)*^T^* is the vector of instantaneous input currents applied to each neuron; the notation [·]^+^ indicates the linear-threshold current to firing rate (*I*/*F*) transfer function [*x*]^+^ = max (*x*, 0); and *W* is the weight matrix of the network. *W* is expressed in units of **A** Hz^−1^ and includes any required current/firing rate (*I*/*F*) gain factors.

##### Homogeneous networks with equal numbers of excitatory and inhibitory neurons.

With the firing rate of each neuron evolving under the dynamics given in Equation 1 above, we define a network weight matrix *W* with dimensions 2*N* × 2*N*, given by the following:

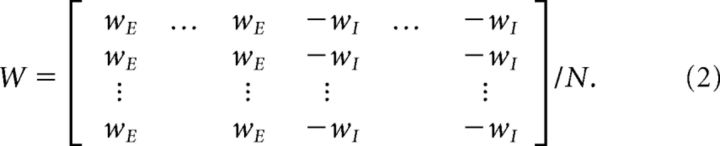
 In this network, the first *N* neurons are excitatory and the subsequent *N* inhibitory, with homogenous all-to-all connectivity. More cortically realistic network structures will be examined below. Neuron gains are assumed to be incorporated into the weight matrix *W*.

##### Stability and fixed-point response analysis.

We examine the fixed points and stability of the network defined in Equation 2 evolving under the dynamics in Equation 1 linearized in the partition where all neurons are active (Hahnloser, 1998; Muir and Cook, 2014). The stability of these networks is determined by examining the eigenvalues and trace of the system Jacobian *J* = (*W* − *I*)/τ, where *I* is the 2*N* × 2*N* identity matrix. Networks of this structure have a trivial eigenvalue (*w_E_* − *w_I_* − 1)/τ = λ_1_/τ. The trace of the Jacobian is given by *Tr*[*J*] = (*w_E_* − *w_I_* − 2*N*)/τ. To guarantee that the network is stable under any finite input (i.e., bounded input/bounded output or BIBO stability), the eigenvalue λ_1_ < 0. We therefore obtain an upper bound on the total weight *w*_E_ provided by each excitatory neuron relative to the strength of inhibition, given by *w_E_* < 1 + *w_I_*. The system trace provides an additional stability constraint *w_E_* < 2*N* + *w_I_*, which for these networks is always a looser bound than that imposed by λ_1_ < 0. For the network to require inhibitory feedback for stability, the excitatory network alone must be unstable; that is, when *w_I_* = 0. This introduces a lower bound on excitatory feedback *w_E_* > 1. For a stable ISN, we therefore obtain the following constraint relating excitation and inhibition:

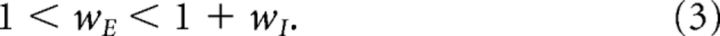
 We analyze the response of the network in steady state, where a constant input is provided and the system allowed to come to rest. The fixed point response of the network is obtained by solving the system dynamics in Equation 1 for the condition **ȧ** = **0** for an input **i**, and is denoted **ā**, x̄ and *ȳ*. For a single neuron *j*, the fixed point is given by the following:

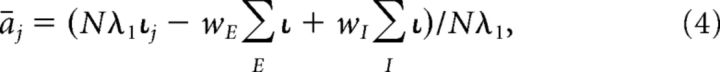
 where ∑*_E_*ι and ∑*_I_*ι denote a summation of the input currents provided to all excitatory or inhibitory neurons, respectively, and λ_1_ = *w_E_* − *w_I_* − 1 as defined above. We also define the eigenvalue with largest real part λ_+_, which can differ from λ_1_ if λ_1_ < 0 in the case of sparse connectivity or in the presence of specific connectivity. For a network to operate in an ISN regime, the excitatory network must be unstable in the absence of inhibition. We define the eigenvalue λ*_E_* as the eigenvalue with largest real part of the excitatory portion of the weight matrix. For an ISN regime to exist, we have the constraint that λ*_E_* > 1.

##### Homogenous networks with unequal numbers of excitatory and inhibitory neurons.

We additionally define networks with varying proportions of inhibitory neurons *f_I_* (Muir and Mrsic-Flogel, 2015). In this work, we examine networks where *f_I_* = 0.2 while maintaining all-to-all nonspecific connectivity (i.e., in the notation of Muir and Mrsic-Flogel, 2015: *h_E_*, *h_I_* = 1; *M* = 1; κ = ∞). In these networks, *N_I_* = *Nf_I_* and *N_E_* = *N*(1 − *f_I_*) denote the number of inhibitory and excitatory neurons, respectively. The connections from each neuron are normalized such that the total output weight from each neuron sums to *w_E_* and *w_I_* for excitatory and inhibitory neurons respectively. Stability and fixed point response analysis are performed following the procedures above.

##### Networks with sparse connectivity.

To generate sparse networks, we followed the procedures in Muir and Mrsic-Flogel (2015). Briefly, fully connected network weight matrices *W* are combined with a sparse *N* × *N* boolean matrix *D*. To generate *D*, the appropriate number of nonzero elements for a column are distributed randomly within each column. This is determined by defining “fill factors” *h*, which specify the proportion of pairwise connections that should exist out of all possible connection partners. The network weight matrix is then given by *W′* = *D* ○ *W*, where ○ denotes the element-wise Hadamard or Schur product and *W′* is renormalized such that columns of *W′* sum to *w_E_* and *w_I_*. In the limit as *N* → ∞, the elements of *D* can be assumed to be independent and therefore are approximated by a Bernoulli distribution. This assumption assists in estimating the eigenvalue spectrum radius of *W′*, described below.

##### Networks with specific excitatory connectivity.

To examine the effect of specific connectivity on the behavior of ISN networks, we defined networks similarly to Muir and Mrsic-Flogel (2015). Briefly, the excitatory network was divided into *M* partitions (“subnetworks”). A proportion *f*_SSN_ ≤ 1 of synapses of each excitatory neuron were reserved to be made with other excitatory neurons within the same subnetwork. The remainder of excitatory synapses were distributed randomly across the entire network with uniform probability. When *f*_SSN_ = 0, no specific connectivity was present and the networks were identical to the homogeneous networks described above. When *f*_SSN_ = 1, excitatory synapses were made exclusively between neurons in the same subnetwork, corresponding to maximally specific connectivity. Connections between excitatory and inhibitory neurons were made without specific connectivity in all cases.

##### Networks with multiple subtypes of inhibitory neurons.

To study the effect of perturbations in networks including multiple inhibitory subtypes, we modeled networks of linear-threshold units consisting of 400 excitatory neurons, 50 parvalbumin (PV)-positive, 25 somatostatin (SOM)-positive (SOM), and 25 vasointenstinal peptide-positive (VIP) inhibitory neurons, with class-specific synaptic connections defined similarly to Litwin-Kumar et al. (2016). We defined the interaction between subpopulations according to the weight matrix *W* as follows:

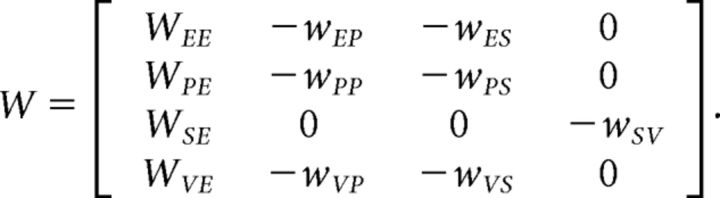
 Note that *W*_Y X_ represents the coupling from subpopulation *X* to *Y*, and E, P, S, and V are the excitatory, PV, SOM, and VIP subpopulations, respectively (cf. Eq. 5 in Litwin-Kumar et al., 2016). As opposed to Litwin-Kumar et al. (2016), in which these weights defined the coupling between single nodes, here they determine the total weight between two subpopulations. Synaptic strength between individual neurons was therefore drawn from a distribution with mean value of *w*_Y__X_ = *W*_Y__X_/*N*_X_, where *N*_X_ is the total number of neurons in the presynaptic subpopulation. The weights in each case were drawn from a zero-truncated Gaussian distribution with mean μ = *w*_Y__X_ and SD σ = 0.2 *w*_Y__X_.

For simulations shown in Figure 9, we chose *W*_EE_ = 1.5 to place the network in the ISN regime and *W*_PE_, *W*_EP_, and *W*_PP_ to have a common value to balance unstable excitation (consistent with dense and strong recurrent connectivity of excitatory ↔ PV neurons as reported experimentally; Hofer et al., 2011). Recurrent coupling between excitatory and SOM neurons (Exc. → SOM and SOM → Exc.) was parameterized by a weight ψ (Exc.–SOM coupling). Other weights were chosen similar to Litwin-Kumar et al. (2016) (cf. their Eq. 7). Our coupling weight matrix was therefore given by the following:

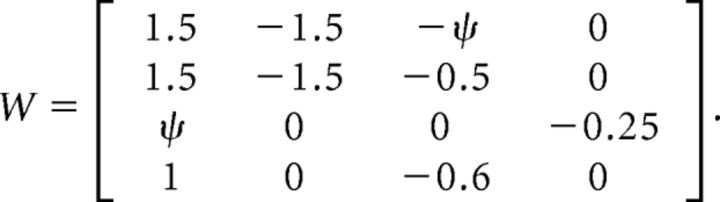


#### Estimating the sparsity of connections in cortex

To estimate realistic parameters for the sparsity of local connections in cortex, we assumed that connections between neurons are made stochastically according to the overlap of simulated axonal and dendritic densities, which are modeled as 2D Gaussian fields. The overlap between two 2D Gaussian fields is proportional to the following:

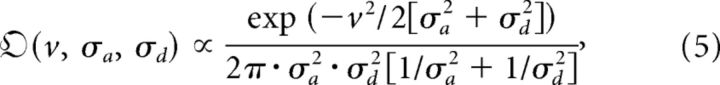
 where *v* is the 2D Euclidean distance between two points and the SDs of axonal and dendritic fields are given by σ*_a_* and σ*_d_*, respectively. Equation 5 is used to compute connection probability fields as a function of axonal and dendritic spreads.

We define the notation 〈·〉_ℝ^2^_ to indicate that the quantity within the brackets should be normalized such that is forms a probability density function over 2D space ℝ^2^; that is, 〈*X*〉_ℝ^2^_ = *X*/∫∫_ℝ^2^_*X*. The synapse formation probability from neuron class *A* to class *B* is then given by the following:

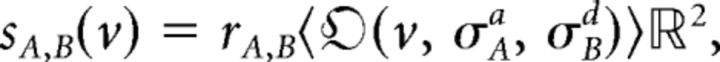
 where *A* and *B* are either *E* or *I* for excitatory and inhibitory, respectively, and *r_A,B_* is the proportion of synapses from class *A* that target class *B*. The factors *r_A,B_* allow us to incorporate class-specific connectivity, which appears to exist in mouse visual cortex in the connections from excitatory to inhibitory neurons (Bock et al., 2011; Bopp et al., 2014).

**Figure 1.**
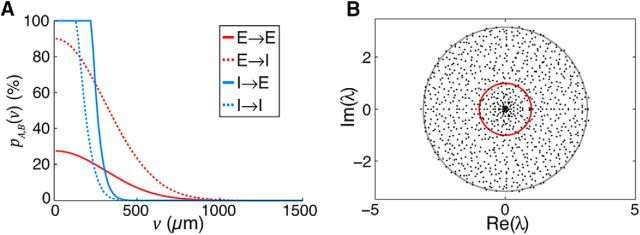
***A***, Simulated connection probability *p_A,B_*(*v*) between neuron classes *E* and *I*. Parameters given in Table 1. ***B***, Eigenvalue spectrum of *W* for a sparse network with *{w_E_, w_I_, h_EE_, h_EI_, h_EI_, h_II_, N}* = {5.4, 56, 0.022, 0.072, 0.084, 0.34, 1000}. The trivial eigenvalue at λ = −8 is not shown. Unit circle (red) and expected bulk radius *q_b_* (gray; Eq. 7) are shown for reference.

We define the expected number of synapses from class *A* to class *B* as *n_A,B_*(*v*) = *S_A_* · *s_A,B_*(*v*), where *S_A_* denotes the number of output synapses from neurons of class *A* (Table 1). The connection probability *p_A,B_* from a neuron of class *A* to a neuron of class *B* at a distance *v* is then given by the following:

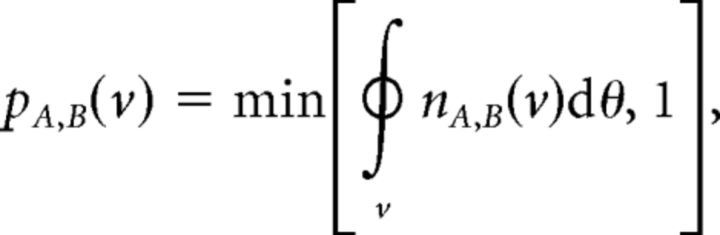
 where 
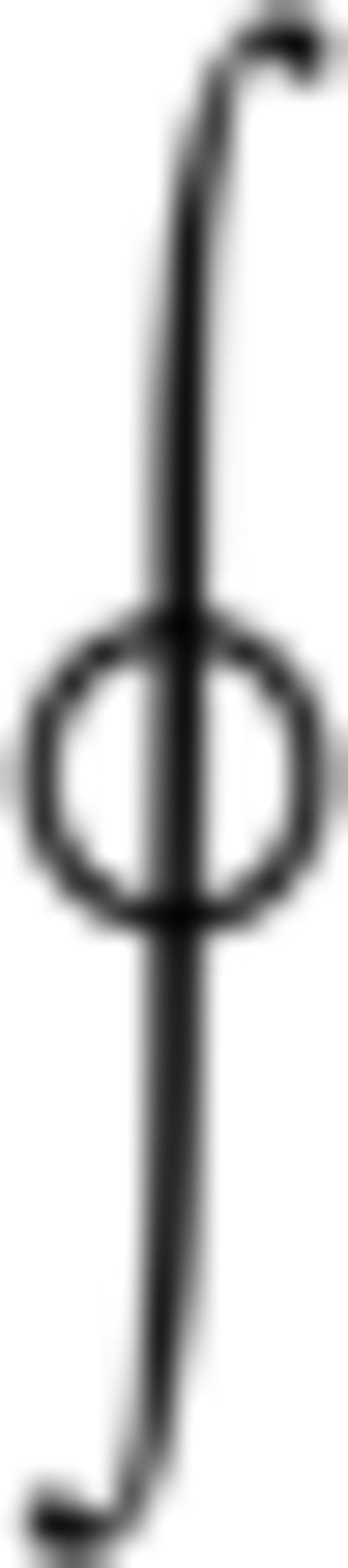
*_v_*dθ indicates integration around an annulus of distance *v* from the origin (Fig. 1). The parameters given in Table 1 result in a proximal *E* → *I* connection probability of *p_E,I_* ≈ 90%, and proximal *E* → *E* connection probability of *p_E,E_* ≈ 25% (Fig. 1).

**Table 1.** Parameters for estimating connection sparsity*^a^*

Parameter		Value	Reference(s)
Axonal width	4σ*_E_^a^*, 4σ*_I_^a^*%	1200 μm, 300 μm	Holmgren et al., 2003; Boucsein et al., 2011; Levy and Reyes, 2012
Dendritic width	4σ*_E_^d^*, 4σ*_I_^d^*%	300 μm, 300 μm	Hellwig, 2000
No. of axonal synapses in L2/3	*S_E_*, *S_I_*	8142, 8566	Binzegger et al., 2004*^a^*
Density of neurons spanning depth of L2/3	η	36,000 mm^−2^	Schüz and Palm, 1989
Proportion of neurons in class A	*f_E_*, *f_I_*	80%, 20%	Gabbott and Somogyi, 1986
Proportion of A → B synapses	*r_E,I_*	45%	Bock et al., 2011; Bopp et al., 2014
	*r_E,E_*	1 − *r_E,I_*	—*^b^*
	*r_I,E_*	1 − *f_I_*	—*^b^*
	*r_I,I_*	*f_I_*	—*^b^*

E, Excitatory; I, inhibitory.

*^a^*Including an estimate of double the number of synapses per neuron in mouse cortex compared with cat cortex.

*^b^*Non-class-specific connectivity.

The sparsity (and equivalently, the fill factor *h*) of connections from class *A* to class *B* is therefore estimated by the following:

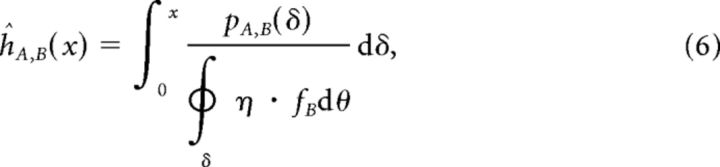
 where Equation 6 should be integrated out to a distance *x* at which the connection probability drops to 0. Taking *x* = 1500 μm for excitatory neurons and *x* = 750 μm for inhibitory neurons, we estimate {*ĥ_EE_*, *ĥ_EI_*, *ĥ_EI_*, *ĥ_II_*} = {0.022, 0.072, 0.084, 0.34}. These low fill factors make the resulting network instances highly unstable for reasonable network size *N* due to expansion of the eigenspectrum bulk, even in the presence of strong inhibitory feedback (Muir and Mrsic-Flogel, 2015; see Fig. 1). The expected radius *q_b_* of the eigenspectrum bulk for a network with class-dependent fill factors is given by the following:

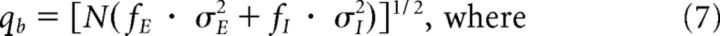


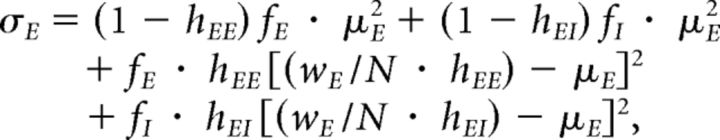


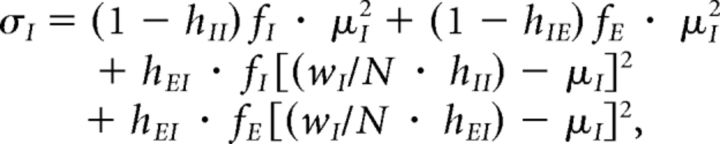
 where μ*_E_* = *w_E_*/*N* and μ*_I_* = *w_I_*/*N* (cf. Muir and Mrsic-Flogel, 2015). To ensure stability in networks with scale smaller than cortex itself, we therefore simulate networks where the radius of the bulk eigenspectrum is controlled by scaling h_*_ by a common factor, such that *q_b_* ≈ 1. Only the connection fill factors h_*_ were modified to increase the fill factor of the weight matrix *W*. The total excitatory and inhibitory weights *w_E_* and *w_I_* were unchanged.

#### Perturbation framework

In general, we introduce a perturbation to a network by defining an input *k*(δ), where *k* defines the input currents to all neurons in a network and δ is a small perturbing effect (δ > 0 corresponds to a positive perturbation in input and δ < 0 corresponds to a negative perturbation). For example:

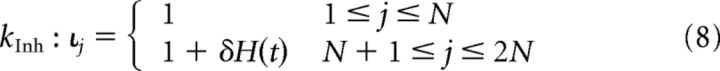
 defines a scheme where all neurons receive a constant input (“1”) and the entire inhibitory population (*N* + 1 ≤ *j* ≤ 2*N*) receives an extra perturbing input δ at *t* = 0. Here, *H*(*t*) is the Heaviside step function.

We assume that a perturbation is made in a network where every neuron is active; inactive subsets of the network can be removed entirely from the system (Hahnloser, 1998; Muir and Cook, 2014). We examine the fixed point **ā** (Eq. 4) of the analytical network, linearized in the state partition when all neurons are active (Muir and Cook, 2014). We assume that the perturbation δ is small enough that no neuron is pushed below threshold.

We assume that a perturbation is only made once the transient response of the network has settled and the network has reached a stable fixed point. We therefore examine the mean-field fixed point response of these networks under the assumption that the effect of stochastic or oscillatory dynamics will be removed by averaging. We likewise neglect the transient effect of a perturbation, and examine only the resulting fixed point response subsequent to the perturbation (i.e., at *t* = ∞).

After a perturbation, we examine the difference between perturbed and unperturbed inhibitory activity *k* : d*ȳ*/dδ under a given perturbation *k*. Generally, we look for a “paradoxical” response of inhibition such that *k* : d*ȳ*/dδ < 0 for δ > 0. For example, under the perturbation of the entire inhibitory population defined in Equation 8 above, the change in inhibitory activity in response to the perturbation is given by the following:

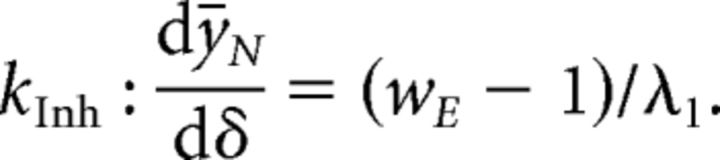
 For this response to the perturbation to meet the characteristics of a paradoxical inhibitory response, we require that d*ȳ_N_*/dδ < 0. Combining this requirement with the conditions for a stable ISN (Eq. 3), we obtain the constraints on network configuration that ensure a paradoxical inhibitory response is observed in a stable ISN. By doing so, we find that the constraints already required by Equation 3 guarantee that a paradoxical inhibitory response will be observed under the global inhibitory perturbation *k*_Inh._ This result implies that a stable ISN will always display a paradoxical response when the entire inhibitory population is perturbed.

##### Perturbation of a single inhibitory neuron.

We examined the other extreme of perturbing a single inhibitory neuron, such that:

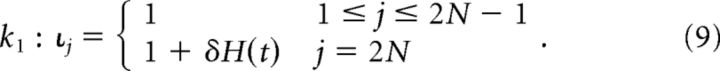
 As before, we computed the change in fixed-point response of a single inhibitory neuron, when that neuron is perturbed, given by the following:

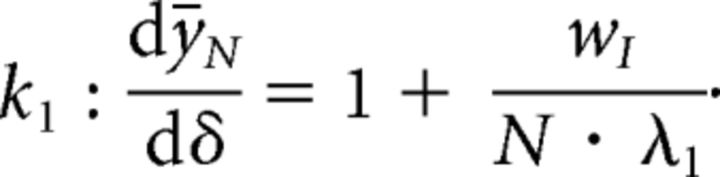
 Under the requirement that a perturbation must lead to a paradoxical response (i.e., *k*_1_ : d*ȳ_N_*/dδ < 0), we find an additional constraint on the excitatory weight *w_E_* > 1 + *w_I_*(*N* − 1)/*N*. This implies that a stable ISN can exhibit a paradoxical effect when a single inhibitory neuron is perturbed if 1 + *w_I_*(*N* − 1)/*N* < *w_E_* < 1 + *w_I_*. We note that (*N* − 1)/*N* → 1 as *N* → ∞, and therefore the range for *w_E_* that satisfies this constraint approaches 0 with increasing *N*.

##### Perturbation of a subset *p* of the inhibitory population.

We investigated the effect of perturbing a subset *p* of the inhibitory population, defined by the following:

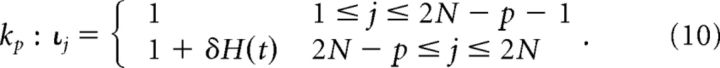
 The derivative of fixed point activity is then given for perturbed inhibitory neurons by the following:

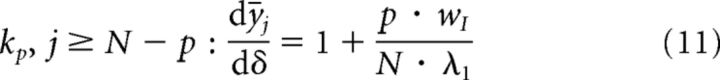
 and for nonperturbed inhibitory and for excitatory neurons by the following:

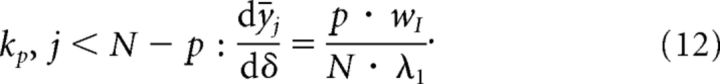
 Under the constraint *k_p_*, *j* ≥ *p* : d*ȳ_j_*/dδ < 0, Equation 11 implies that at least a proportion *p*/*N* > −λ_1_/*w_I_* of the inhibitory population must be perturbed to observe a paradoxical effect in the perturbed neurons.

Note that, in networks with different numbers of excitatory and inhibitory neurons, the proportions were measured as a fraction of the actual number of inhibitory neurons in the network, *p*/*N_I_*. Perturbations were performed identically in networks with sparse recurrent synaptic connectivity.

##### Perturbation of networks with specific excitatory connectivity.

In networks with specific connectivity within excitatory subnetworks, we investigated whether subnetwork-specific activation patterns changed the behavior of a network in response to an inhibitory perturbation. Perturbations under global network activity were performed as in Equation 10. Under subnetwork-specific activation, we defined a perturbation as follows:

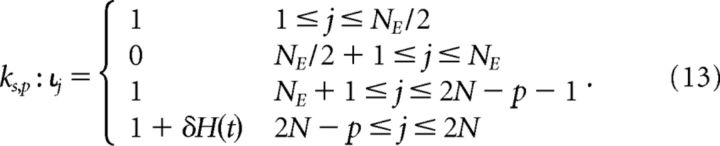
 Here, the excitatory neurons in the subnetworks comprising half of the excitatory population (i.e., 1 ≤ *j* ≤ *N_E_*/2) receive an external input drive, whereas neurons in the second half of the excitatory population (i.e., *N*_E_/2 + 1 ≤ *j* ≤ *N_E_*) receive no external input. A subset *p* of the inhibitory population receives a perturbation, as before.

##### Perturbation by injecting a global inhibitory current.

We examined the effect of perturbing the entire network by injecting a global inhibitory current, as might be produced by infusing cortex with a GABA agonist. The perturbation is defined by *k_g_* : ∀ *j*, ι*_j_* = 1 + δ*H*(*t*). The derivative of fixed point activity for all neurons is then given by the following:

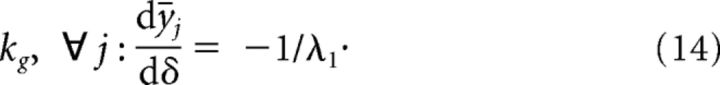
 Because *k_g_* : d*ȳ_j_*/dδ is always positive for a stable ISN (i.e., satisfying Eq. 3), no paradoxical response of inhibitory neurons is possible under the network-global perturbation *k_g_*.

##### Perturbation by modifying inhibitory weight w_I_.

Alternatively, infusion of GABA agonists or antagonists might result in an divisive rather than subtractive effect on inhibitory input currents. We therefore computed the change in fixed point response d*ȳ_j_*/d*w_I_* when the total inhibitory weight *w*_I_ is perturbed, requiring that, for an increase in inhibitory weight, the paradoxical response would be for the inhibitory network to increase its activity: that is, d*ȳ_j_*/d*w_I_* > 0. We define the input to the network as follows:

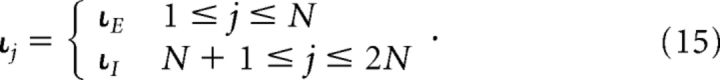
 The fixed point response of the network under this input is given by the following:

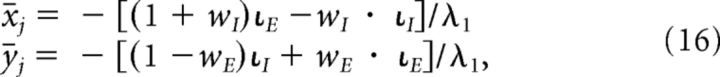
 and the resulting change in fixed point response by the following:

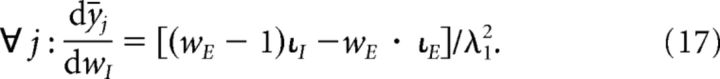
 For a stable ISN, a regime exists such that if the inputs to excitatory and inhibitory neurons differ (i.e., ι*_E_* ≠ ι*_I_*), then the paradoxical response ∀ *j* : d*ȳ_j_*/d*w_I_* > 0 is evoked when ι*_I_* > *w_E_* · ι*_E_*/(*w_E_* − 1). Unfortunately, this regime only occurs when ∀ *j* : *x̄_j_*, *ȳ_j_* < 0; that is, when the network is silenced.

##### Perturbation in networks with multiple subtypes of inhibitory neurons.

We perturbed varying fractions of either the PV subpopulation alone or in conjunction with other inhibitory subpopulations. When perturbing more than one inhibitory class, the same fraction of neurons was perturbed in the appropriate subpopulations. We then measured the critical fraction of inhibition needed to be perturbed to see the paradoxical effect, as for networks with a single inhibitory class. The paradoxical effect was assayed by an increase in the average activity of perturbed PV neurons in response to negative inhibitory perturbations (i.e., δ < 0).

#### Spatial perturbation model

We simulated inhibitory perturbations in a neural field model with spatial extent. Two plates of simulated nodes were constructed, corresponding to excitatory and inhibitory fields on a 2D torus. Each node evolved under the dynamics in Equation 1. The weight matrix *W* was constructed using wrapped Gaussian axonal and dendritic fields as follows:

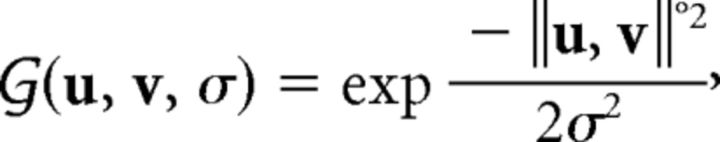
 where ‖**u**, **v**‖^°^ is the Euclidean distance between node locations **u** and **v** on a 2D torus space *T*^2^ and σ is the SD of the field. The neural field was defined to span **f** = 2400 × 2400 μm, with a simulation resolution of 33 μm per mesh point. The “width” of a field was defined as 4σ. Individual weights *w*_ji_ ϵ *W* between neurons *i* and *j* were given by the product:

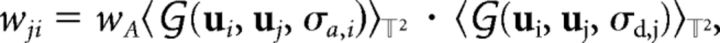
 where4σ*_i_^a^* and 4σ*_j_^d^* define the axonal and dendritic fields widths of neurons *i* and *j*; the notation 〈·〉_
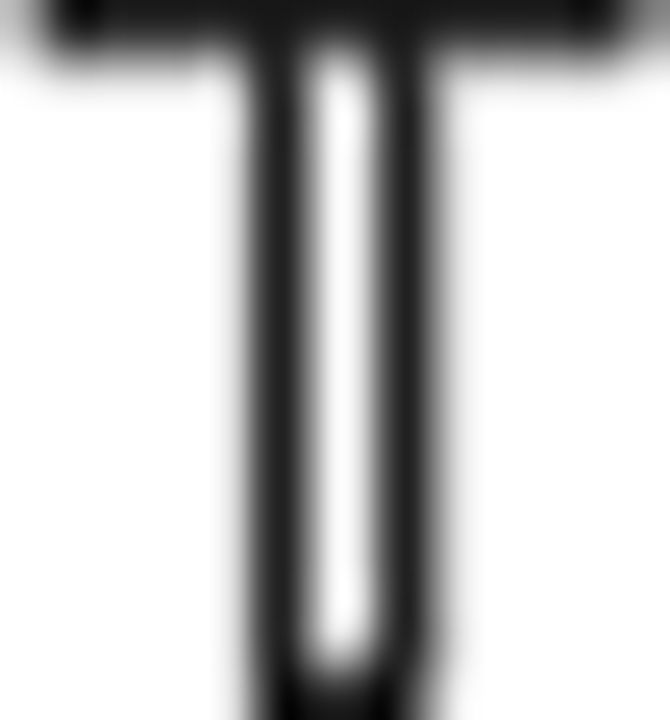
^2^_ defines a probability density function as above; and *w_A_* defines the total synaptic weight from neurons of class *A*. Other parameters for the spatial perturbation model are given in Table 1.

We simulated activity of the spatial model under a constant input ∀ *_j_* : ι*_j_* = 1 for *t* = (−10, 0). A subset of inhibitory nodes were subsequently perturbed under the following:

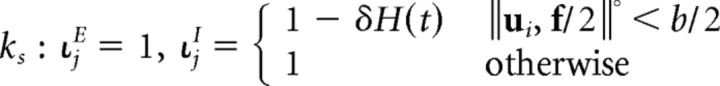
 where *b* is the diameter of the spatial perturbation, within which all inhibitory nodes are perturbed; **f**/2 is the center of the simulated field; and δ is the strength of the perturbation as defined previously. The perturbation stimulus was applied for *t* = (0, 10). The perturbation was simulated for a range of *b* = (50 μm, 400 μm) in 20 steps and linear interpolation was used to estimate the threshold perturbation diameter, *b̂_p_*.

#### Comparison with optogenetic perturbation results from Atallah et al. (2012)

Atallah et al. (2012) used optogenetic activators and inhibitors expressed selectively in PV-positive inhibitory neurons to perturb inhibitory activity in mouse visual cortex (Atallah et al., 2012). They recorded responses to visual stimuli of varying contrast in the presence of optogenetically induced inhibitory suppression and activation while recording inhibitory and excitatory synaptic input currents impinging on excitatory neurons. In response to inhibitory suppression using ARCH, PV inhibitory neurons reduced their activity by 40% and excitatory neurons increased their activity to 140% of baseline (see their Fig. 2*D*,*F*). In response to inhibitory activation using ChR2, PV inhibitory neurons increased their activity to 120% of baseline and excitatory neurons reduced their activity by 30% (see their Fig. 2*E*,*G*). For comparison with these results, we found combinations of network and perturbation parameters in our simulated networks that resulted in similar perturbations of inhibitory and excitatory activity observed by Atallah et al. (2012) (see our Fig. 8). We included uncertainty of 20% around each target change in excitatory and inhibitory activity.

We defined a simulated excitatory neuron as displaying a paradoxical response if the result of an inhibitory perturbation was to shift the net inhibitory input current by at least 10% of its unperturbed value.

#### Spiking networks with conductance-based neurons

##### Neuron model.

Spiking neurons were modeled using an exponential integrate-and-fire model (Brette and Gerstner, 2005), without adaptation. The dynamics of the membrane potential *V_m_*(*t*) of a single model neuron evolved under the following equation:

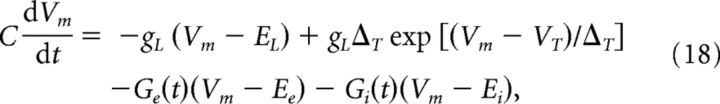
 where *C* is the membrane capacitance, *g_L_* is the leak conductance, and *E_L_* is the resting potential. The exponential term describes the activation of sodium current. The parameter Δ*_T_* is called the slope factor and *V_T_* is the threshold potential. Once the membrane potential *V_m_* reaches the threshold *V_T_*, a spike is emitted and the membrane potential is reset to a fixed voltage, *V*_reset,_ for a refractory period *t*_ref._

*E_e_* and *E_i_* are the reversal potentials for excitation and inhibition, respectively. *G_e_*(*t*) and *G_i_*(*t*) represent the total excitatory and inhibitory conductances at time *t*, given by the following:

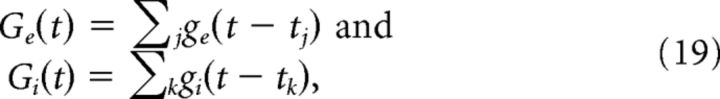
 where the times of occurrence of excitatory and inhibitory synaptic events are denoted by *t_j_* and *t_k_*, respectively. *g_e_* and *g_i_* denote the membrane conductance changes elicited by a single excitatory or inhibitory synaptic event, which are modeled as α-functions, given by the following:

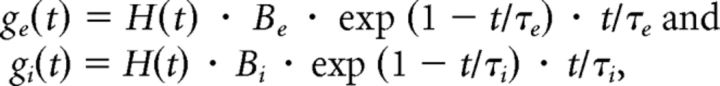
 where *B_e_* and *B_i_* denote the peak excitatory and inhibitory synaptic conductances, respectively. The integral of the conductances is given by the following:

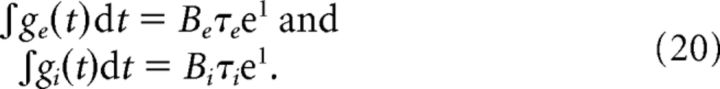
 In these equations, e^1^ = exp(1) = 2.718. The default parameters of the neuron model are listed in Table 2. Default values of peak synaptic conductances were *B_e_* = 0.1 nS, *B_i_* = 0.2 nS, and τ*_e_* = 1 ms, τ*_i_* = 1 ms. Note that the effective time constant of the synapses, defined as the time from a spike until the synaptic current decays to the 37% of the peak current, is much longer (τ_eff_ > 3 ms for τ = 1 ms). To simulate the spiking networks, we used NEST software (Gewaltig and Diesmann, 2007). The implementation uses a fourth-order Runge–Kutta–Fehlberg solver with adaptive step size to integrate the differential equation.

**Table 2.** Parameters of the spiking neuron model

Parameter		Value
Membrane capacitance	*C*	120 pf
Leak conductance	*G_L_*	7.14 nS
Resting potential	*E_L_*	−70 mV
Threshold voltage	*V_T_*	−50 mV
Reset voltage	*V*_reset_	−60 mV
Reversal potential	*E_e_*, *E_i_*	0 mV, −75 mV
Synaptic time constant	τ*_e_*, τ*_i_*	1 ms
Slope factor	Δ*_T_*	2 ms
Refractory period	*t*_ref_	2 ms

##### Network simulations.

Networks were composed of *N_E_* excitatory and *N_I_* inhibitory neurons. Excitatory and inhibitory neurons had the same properties and parameters as described above. All neurons received a baseline input. This was modeled as an independent homogeneous Poisson process with firing rate *r_b_*. The strength of synaptic connectivity is parameterized by the peak synaptic conductance, which was denoted as *B_b_* for the baseline input. Connection delays were chosen as the fixed value of *d* for the input synapses; synaptic delays for recurrent connections were drawn from a random exponential distribution with mean *d*.

Recurrent connections were drawn from a binomial distribution. The mean connection probability from the presynaptic subpopulation *X* ϵ {*E*,*I*} to postsynaptic subpopulation *Y* ϵ {*E*,*I*} was *C_X→Y_*. The connection weights between established connections were drawn from a truncated Gaussian distribution with a mean of *B_X→Y_* and SD of *B_X_*_→*Y*_/5. The mean value for E → E and E → I connections were set to *B_E→E_* = *B_E→I_* = *B_e_*; similarly, the mean value for I → E and I → I connections were set as *B_I→E_* = *B_I→I_* = *B_i_*. The parameter space for the balance of excitation and inhibition in the network is scanned by changing these two parameters (e.g., in Fig. 10*D*).

The stimulation protocol of the network comprised three phases: an initial transient phase where the spiking activity was not analyzed (*T*_trans_); the baseline duration where the normal activity of the network was recorded (*T*_base_); and the perturbation period during which a certain fraction of the inhibitory population was perturbed (*T*_pert_). To obtain reliable estimates of firing rates, simulated perturbations were repeated for *N*_trial_ trials, with each trial lasting for *T*_trial_ = *T*_trans_ + *T*_normal_ + *T*_pert._ The default parameters of network simulations are listed in Table 3.

**Table 3.** Parameters of the spiking network simulations

Parameter		Value
No. of neurons	*N_E_*, *N_I_*	1600, 400
Connection probability	*C_E→E_*, *C_E→I_*, *C_I→E_*, *C_I→I_*	15%, 15%, 100%, 100%
Baseline input	*r_b_*	9.6 kHz
Strength of baseline input	*B_b_*	0.1 nS
Average synaptic delay	*d*	0.1 ms
Simulation time (transient, baseline, perturbation)	*T*_trans_, *T*_base_, *T*_pert_	0.15 s, 0.5 s, 0.5 s
No. of trials	*N*_trial_	5 (Fig. 10*D*) or 10 (Fig. 10*A*–*C*)
Strength of input perturbation	δ	0.4 kHz

The perturbation was performed by reducing the baseline input to *p* inhibitory neurons by δ = 0.4 kHz (i.e., by ∼4%) and was repeated for a range of inhibitory fractions *p*/*N_I_* = {0.1,0.25,0.5,0.75,1}. For each perturbation, the mean firing rates of each subpopulation (excitatory, non-perturbed inhibitory and perturbed inhibitory) in the normal state (*r*_base_) and during perturbation (*r*_pert_) were computed by averaging over time, trials, and the subpopulation. The change in the firing rate due to perturbation was then computed as *r*_diff_ = *r*_pert_ − *r*_base._ Because the perturbation is performed by decreasing the input to a fraction of inhibitory subpopulation, a positive *r*_diff_ for the perturbed inhibitory fraction implies the existence of the paradoxical inhibitory response. We estimated the minimum fraction of inhibition to see this paradoxical effect for a given network (i.e., the value of *p*/*N_I_* such that *r*_diff_ = 0) by linearly interpolating *r*_diff_.

##### Mean-field approximation.

The mean-field analysis of the network dynamics was performed by analyzing the average behavior of the network. Let *r_e_* and *r_i_* denote the mean rates of the excitatory and inhibitory populations within a network. Combining Equations 19 and 20, the temporally averaged excitatory and inhibitory conductances input to an example neuron can be written as follows:

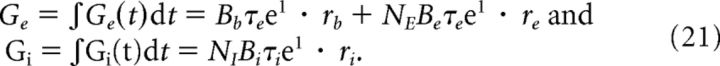
 The total excitatory conductance *G_e_* is composed of two terms: the baseline external input and recurrent input from presynaptic excitatory neurons. The inhibitory conductance *G_i_* results from presynaptic inhibitory neurons in the network.

To obtain the effective change in the membrane potential as a result of these input conductances, we must consider the effective drives from Equation 18:

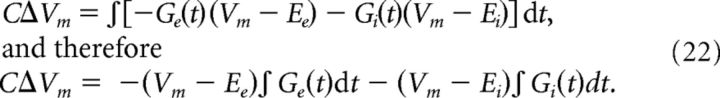
 Here, we have made a simplifying assumption that the population average membrane potential of the network is constant and can be approximated by the time-averaged membrane potential of the network, denoted by *V_m_*. Substituting Equation 21 into Equation 22, we obtain the effective change in membrane potential *V*_tot,_ given by the following:

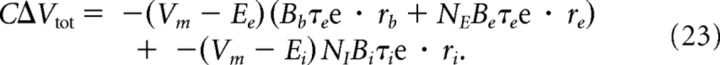
 Note that the effective input is similar for any neuron independent of its subtype identity (excitatory or inhibitory). Furthermore, we make the ansatz that the rates of excitatory and inhibitory subpopulations are the same: *r_e_* = *r_i_* = *r*. This is based on the fact that both subtypes have the same single-cell parameters and network connectivity profiles and the input to both subnetworks is similar in the unperturbed state. Due to this homogeneity, they have the same mean firing rates. Equation 23 can therefore be further simplified to the following:

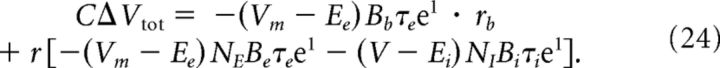
 The first term on the right side is a constant external input and the second term is the recurrent input as a function of the average firing rate *r* of the entire network. Both terms depend on the average membrane potential *V_m_*.

We make a final assumption that the firing rate of a neuron depends linearly on its input (linear input–output transfer function). We take this linear dependence to be *r*_out_ = Δ*V*_inp_/θ, where θ = *V_T_* − *V*_reset_ is the difference between the reset voltage *V*_reset_ and the threshold voltage *V_T_*. Equation 24 can be rewritten as a self-consistent mean-field equation given by the following:

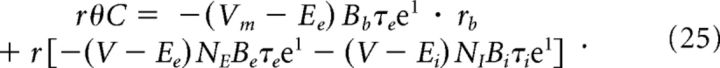
 By defining the total baseline input as *s_b_* = −(*V_m_* − *E_e_*)*B_b_*τ*_e_*e^1^ · *r_b_*/θ*C* and the total recurrent weight as *w* = [ −(*V_m_* − *E_e_*)*N_E_B_e_*τ*_e_*e^1^ − (*V_m_* − *E_i_*)*N_I_B_i_*τ*_i_*e^1^]/θ*C*, we obtain *r* = *s_b_* + *w* · *r* and therefore *r* = *s_b_*/(1 − *w*). The stability of the linearized system can be ensured by constraining the total recurrent weight by *w* < 1. For the full network, this provides a condition for stability, given by the following:

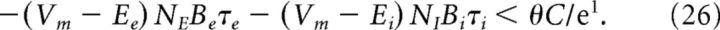
 Note that because the left side of Equation 26 depends on the average membrane potential *V_m_* of the network, the condition can be evaluated at different “operating points.” The stability of the excitatory subnetwork in the absence of the inhibitory subnetwork can be examined by setting the recurrent inhibitory contribution to 0 in Equation 26. This provides a constraint that ensures the network requires inhibitory feedback for stability, given by −(*V_m_* − *E_e_*)*N_E_B_e_*τ*_e_* ≥ θ*C*/e^1^; we therefore obtain the following constraint:

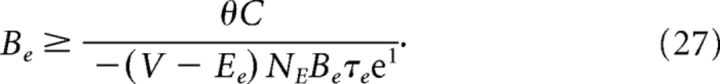
 This constraint is plotted as the vertical line denoting the boundary between the ISN and non-ISN regimes in Figure 10*D*.

#### Experimental design and statistical analysis

No statistical testing was performed. Models and simulations to reproduce all results herein are available from FigShare (DOI https://doi.org/10.6084/m9.figshare.4823212).

## Results

**Figure 2.**
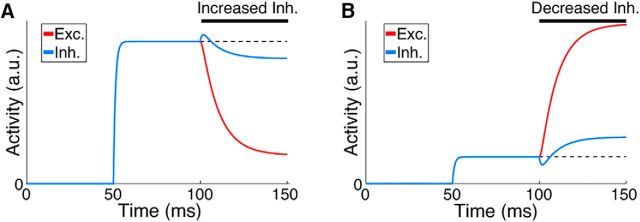
Globally perturbing the inhibitory network gives rise to a paradoxical inhibitory response in ISNs. ***A***, ***B***, Effect on activity of excitatory and inhibitory neurons in an ISN model of 100 fully connected firing rate neurons of increasing (***A***) and decreasing (***B***) excitatory input to the inhibitory population (see Materials and Methods). At 50 ms input is injected to all neurons. At 100 ms, only the input to the inhibitory population is perturbed. Note that increasing the inhibitory input results in a counterintuitive decrease in overall inhibitory activity and vice versa. Parameters: {*w_E_*, *w_I_*, τ} = {5, 20, 10 ms}. Dashed line is shown for reference to preperturbation activity.

Simple ISNs display counterintuitive dynamics when inhibitory activity is perturbed by increasing or decreasing excitatory input into inhibitory neurons. If inhibition is reduced by removing input then the network effect is to increase the activity of inhibitory neurons (Fig. 2*A*); conversely, if extra input is provided to inhibitory neurons, then the network responds by decreasing their activity (Fig. 2*B*). This has been termed the “paradoxical” inhibitory response (Tsodyks et al., 1997) and arises through nonlinear network dynamics introduced by unstable excitatory feedback. This counterintuitive effect of perturbing inhibition has been put forward as a signature of ISN dynamics that could be detected in cortical networks (Tsodyks et al., 1997). This is an experimentally accessible metric because neurons are often being recorded and activated at the same time. When the entire inhibitory population of an ISN is perturbed simultaneously, then the paradoxical effect emerges, as shown in Figure 2. However, under typical experimental conditions, only a fraction of the inhibitory population can be perturbed. This raises the question of whether the paradoxical effect will be observed if only portions of the inhibitory population are perturbed. Recent results based on direct activation and suppression of the inhibitory network (Atallah et al., 2012) did not reveal evidence for a paradoxical inhibitory response. Based on these results, some investigators have inferred that an ISN regime may not exist in the superficial layers of mouse visual cortex (Litwin-Kumar et al., 2016). It remains unclear whether experimental methods for perturbing inhibition will be sufficient to reveal a signature of ISN dynamics.

### Perturbations in networks of firing rate neurons in ISN and non-ISN regimes

To explore the properties of ISNs and non-ISNs and to investigate how they respond to perturbations over a wide range of parameters, we first developed a simple analytically tractable model of a cortical network. For this, we used nonspiking linear-threshold neuron models because they provide a good approximation to the *F–I* curves of adapted cortical neurons (Ermentrout, 1998). Networks were built using homogeneous synaptic connectivity and equal numbers of excitatory and inhibitory neurons (see Materials and Methods). In these models, we simulated synaptic inputs by injecting currents proportional to presynaptic activity.

We analyzed the stability and dynamic properties of this network model to determine the conditions under which it operates in an ISN regime. The stability of networks was determined by expressing all synaptic connections between pairs of neurons as a weight matrix *W* and then analyzing the properties of this matrix. Each network has an associated property known as the trivial closed-loop eigenvalue λ_1_, which depends on the strength of excitation and inhibition within the network and the dynamical properties of the network (see Materials and Methods). If this value is large (i.e., λ_1_ > 0), then the network can become unstable. This is because a pattern of activity in the network can become amplified through local recurrent feedback and the firing activity of the neurons involved could increase without bound. Alternatively, if λ_1_ ≤ 0, then the activity of all neurons in the network is guaranteed not to increase without bound; this is defined as a stable network.

For a network to operate in an ISN regime, the network must be unstable in the absence of inhibition yet stable with inhibitory feedback (Tsodyks et al., 1997). By setting the synaptic strength of inhibition *w_I_* to 0, we found that the excitatory network is unstable (i.e., the largest real eigenvalue of the excitatory portion of the weight matrix λ*_E_* > 1) when the total recurrent synaptic weight contributed by a single excitatory neuron is >1; that is, *w_E_*(1 − *f_I_*) > 1. The interpretation of this value for recurrent excitatory weight is that, in an active excitatory network with no inhibition, a single spike from an excitatory neuron leads to at least one extra spike in the rest of the network on average (i.e., open-loop excitatory gain >1).

To ensure stability in the entire network (i.e., λ_1_ ≤ 1 in the presence of inhibitory feedback), we found a constraint relating the strength of excitation and inhibition that guarantees local inhibition is strong enough to keep recurrent excitation in check. For networks operating in the ISN regime, the relative strengths of excitation and inhibition must satisfy 1 < *w_E_* < 1 + *w_I_* (Eq. 3).

#### Perturbation of entire inhibitory population

For small networks consisting of a single excitatory and a single inhibitory neuron (Tsodyks et al., 1997; Litwin-Kumar et al., 2016), perturbing the inhibitory neuron will always result in a paradoxical response in an ISN. We considered whether this result holds true for larger networks with many excitatory and inhibitory neurons. We began by estimating the effect of a perturbation to the entire inhibitory population on the activity of a single inhibitory neuron (Eq. 8). We ignored any transient effect of a perturbation, comparing only the steady-state response of a network before and after the perturbation (see Materials and Methods; Figure 2).

For the paradoxical effect to appear, a positive perturbation provided to the inhibitory population must result in a counterintuitive reduction in the activity of the inhibitory neuron under measurement. To determine whether this “paradoxical” effect occurs for a given network and given perturbation, we calculated the change in firing rate of a chosen inhibitory neuron with respect to a perturbation (see Materials and Methods).

For a stable ISN as defined above (Eq. 3; see Materials and Methods), we found that a global perturbation of the inhibitory population will always evoke a paradoxical effect. This result shows that the dynamics of our large networks are comparable to previous simplified ISN models (Tsodyks et al., 1997; Litwin-Kumar et al., 2016).

#### Perturbation of a single inhibitory neuron

Because not all inhibitory neurons within a cortical region will be perturbed with electrophysiological or optogenetic approaches under realistic experimental conditions, we investigated how networks respond when only a fraction of the inhibitory neurons are perturbed. Starting with the extreme case of perturbing a single inhibitory neuron (Eq. 9), we found that a narrow range of excitatory synaptic strength *w_E_* exists, within which the paradoxical effect can be evoked (see Materials and Methods). However, the range for *w_E_* that satisfies this constraint shrinks rapidly to 0 as the size of the network increases, making this regime unlikely to exist in cortex.

#### Perturbation of a subset *p* of the inhibitory population

We then investigated the effect of perturbing a larger subset of the inhibitory population, as is likely to be the case under experimental conditions. We injected a positive or negative current into *p* inhibitory neurons (see Materials and Methods; Eq. 10). We found that, for networks in a stable ISN regime, the relative total synaptic strength of excitatory and inhibitory neurons determines a minimum proportion *p*/*N* > −λ_1_/*w_I_* of the inhibitory network that must be perturbed to observe a paradoxical response in the perturbed neurons.

**Figure 3.**
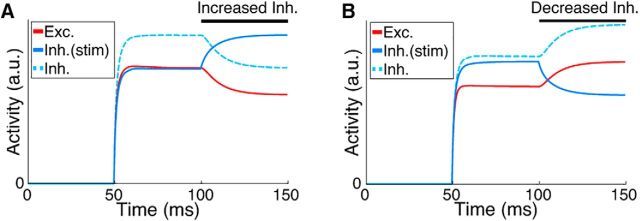
Perturbing only a proportion of the inhibitory population may not give rise to a paradoxical inhibitory response. ***A***, ***B***, Result of increasing (***A***) and decreasing (***B***) input to a portion *p* = 50% of the inhibitory population (cf. Fig. 2). Although this network is an ISN with same parameters as in Figure 2, the response of inhibitory neurons to perturbation is starkly different. No evidence for the paradoxical response is visible, the perturbed inhibitory neurons simply follow the perturbing stimulus. Dashed trace is the response of nonstimulated inhibitory neurons shifted up for visibility. The response of excitatory neurons (red) and nonstimulated inhibitory neurons (dashed) are identical.

**Figure 4.**
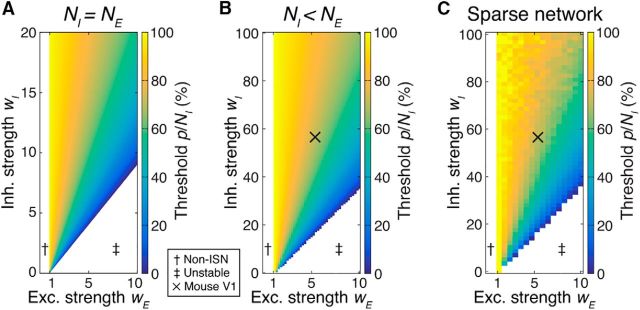
Many inhibitory neurons must be perturbed to evoke a paradoxical inhibitory response. ***A***, Minimum proportion of the inhibitory population *p*/*N* that must be perturbed for the paradoxical effect to appear in the perturbed neurons in a network with equal numbers of excitatory and inhibitory neurons. This analytical result does not depend on the size of the network *N*. Parameters: {*h_I_*, *h_E_*, *f_I_*, τ} = {1, 1, 0.5, 10 ms}. ***B***, Miminum proportion of inhibition *p*/*N_I_* for a network with *f_I_* = 20%. Other parameters: {*h_I_*, *h_E_*, τ, *N_E_*, *N_I_*} = {1, 1, 10 ms, 80, 20}. Note the difference in scale compared with ***A***. ***C***, Minimum proportion of the inhibitory population *p*/*N* that must be perturbed under for the paradoxical effect for networks with sparse synaptic connectivity between excitatory and inhibitory neurons. Note that this does not affect the overall trend for averaged response of stimulated inhibitory neurons (cf. ***B***), but the stochastic effect of introducing sparse connections in smaller networks is evident. Parameters: {*h_EE_, h_EI_, h_IE_, h_II_, N_E_, N_I_*} = {0.1, 0.5, 0.5, 0.5, 4000, 1000}. “X” in ***B*** and ***C***, estimated nominal parameters for mouse visual cortex {*w_E_, w_I_*} = {5.4, 56}. This estimate gives *p*/*N_I_* = 70%. †Non-ISN regime; ‡unstable regime.

If a smaller proportion of the inhibitory network is stimulated, then the paradoxical response does not occur in either the perturbed or nonperturbed inhibitory neurons (Fig. 3). Depending on the operating regime of the network, the proportion of inhibitory neurons that must be perturbed can be considerable, approaching 100% (Fig. 4). Importantly, this proportion does not depend on the size of the network *N* or on the strength of a perturbation δ.

#### Perturbation by injecting a global inhibitory current

Some experimental perturbations, such as infusion of neurotransmitters or chemical agonists of inhibition, result in injection of inhibitory currents across the entire network (i.e., in both inhibitory and excitatory neurons). We therefore examined the case of such a global perturbation in our models (see Materials and Methods; Eq. 14). We found that this mode of perturbation cannot elicit a paradoxical inhibitory response in a network operating in a stable ISN regime. Experimental methods that modulate inhibitory inputs to all neurons globally as opposed to perturbing the inhibitory population alone cannot therefore be used to demonstrate an ISN regime in cortex.

#### Perturbation by modifying effective inhibitory synaptic strength

It is possible that some experimental perturbations, such as infusion of a GABA antagonist, may result in a divisive rather than subtractive effect on inhibitory input. We investigated the effect of divisive perturbations by scaling the effective inhibitory synaptic strength *w_I_*. We computed the change in neuronal responses when effective inhibitory synaptic strength is perturbed, requiring that, for an increase in inhibitory synaptic strength, the analogous “paradoxical” response would be for the inhibitory network to increase its activity (see Materials and Methods; Eq. 15). We provided a constant but different input current to excitatory and inhibitory neurons, ι*_E_* and ι*_I_*, respectively.

We found that, for a network operating in a stable ISN regime, there is no combination of relative excitatory and inhibitory input or synaptic weight that can give rise to a paradoxical inhibitory response when the inhibitory synaptic strength is perturbed. This result implies that global modulation of inhibitory weights or other similar divisive modulation of inhibition cannot be used to demonstrate an ISN regime in cortex.

#### Networks with realistic proportions of excitatory and inhibitory neurons

The networks described above have equal numbers of excitatory and inhibitory neurons, similar to classical ISN networks. However, in mammalian cortex, ∼20% of neurons are inhibitory (Gabbott and Somogyi, 1986). We therefore redefined our network according to Muir and Mrsic-Flogel (2015) and set the proportion of inhibitory neurons in the network to 20% while maintaining all-to-all nonspecific connectivity. We computed numerically the proportion of the inhibitory population that must be stimulated to observe the paradoxical effect in the stimulated neurons (Fig. 4*B*; see Materials and Methods). In general, networks with fewer inhibitory neurons are less stable. Indeed, an increase in *w_I_* is required for stability (cf. Fig. 4*A*,*B*; note the different scales of inhibitory strength in *A* and *B*). However, we observed the same trends for evoking a paradoxical inhibitory response in networks with fewer inhibitory neurons, as for the networks with equal numbers of excitatory input.

#### Sparse connectivity does not affect the proportion of inhibition that must be perturbed

Synaptic connections between neurons in the neocortex are not all-to-all; neurons connect to their immediate neighbors with an average probability of only ∼20% for recurrent excitatory connections (Gabbott and Somogyi, 1986). Connections between neighboring inhibitory and excitatory neurons are much more dense, with close to 100% connection probability between neighboring excitatory and PV-positive inhibitory neurons (Bock et al., 2011; Fino and Yuste, 2011; Hofer et al., 2011; Martin, 2011; Bopp et al., 2014), but connection probabilities fall off dramatically with distance (Boucsein et al., 2011; see Materials and Methods).

To examine the effect of sparse connectivity, we expanded upon the work in Muir and Mrsic-Flogel (2015) by introducing connection sparsity parameters that describe the number of synaptic connections made between nearby neurons as a proportion of all possible partners. We estimated separate sparsity parameters for recurrent excitatory, excitatory → inhibitory, inhibitory → excitatory, and recurrent inhibitory connections based on the assumption of stochastic connections formed between neurons with overlapping axonal and dendritic arbors and to match reported connection probabilities (Peters' rule; see Materials and Methods; Peters, 1979; Reimann et al., 2015).

By computing the proportion *p*/*N_I_* of the inhibitory population that must be stimulated to observe the paradoxical effect, we found that, if one records the average response of stimulated inhibitory neurons, then *p*/*N_I_* only differs from the fully connected network in terms of stochasticity induced by the random sparsity structure of individual instances of *W* (Fig. 4*C*). Estimates for nominal parameters of total synaptic strength in rodent cortex are indicated by “X” in Figure 4, *B* and *C*, suggesting that ∼70% of inhibitory contribution must be perturbed to observe the paradoxical inhibitory response in cortex. However, due to the spatial dependence of connectivity and the tendency for local inhibition to be strong, dense, and class-specific (Bock et al., 2011; Fino and Yuste, 2011; Hofer et al., 2011; Martin, 2011; Bopp et al., 2014), inhibition may be even stronger than this estimate, which is based on uniform connection probabilities. Our results predict that a large fraction of inhibitory neurons must be perturbed to evoke a paradoxical response in cortex.

### Perturbations in networks with specific excitatory connectivity

**Figure 5.**
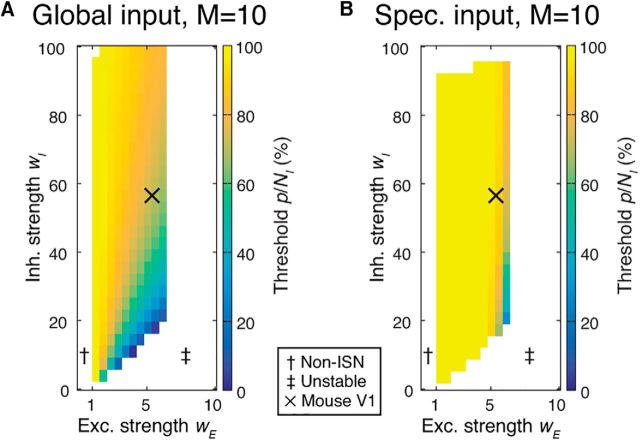
Inhibitory perturbations in networks with specific excitatory connectivity. ***A***, Minimum proportion of inhibition that must be perturbed to observe a paradoxical response is unchanged by the presence of *M* excitatory subnetworks (*M* = 10; other parameters as in Fig. 4*B*), under global external input (cf. Fig. 4*B*). Specific excitatory connectivity leads to instability for excitatory synaptic strength *w_E_* stronger than ∼6.5. ***B***, When external input is provided instead to half of the excitatory subnetworks (Eq. 13), larger fractions of inhibition must be perturbed. Conventions are as in Figure 4. Estimate at “X” in ***A*** corresponds to *p*/*N_I_* = 70%; in ***B***, 90%.

In previous sections, we examined networks in which local excitatory connections were made sparsely, but with identical probability between all excitatory neurons. However, pairwise excitatory connectivity is modulated by neuronal response similarity in both rodent noncolumnar visual cortex (Ko et al., 2011; Cossell et al., 2015) and in columnar visual cortex (Malach et al., 1993; Bosking et al., 1997; Muir et al., 2011; Martin et al., 2014). The dynamics of inhibitory perturbations in stabilized networks with this structure has not been examined. We therefore considered that the presence of strongly coupled excitatory subnetworks might affect network responses to inhibitory perturbation. In addition, the impact of inhibitory perturbations may depend on whether the external network drive during a perturbation is random or is subnetwork specific, such as by stimulating with high contrast oriented gratings in visual cortex. We therefore studied perturbations in networks where excitatory neurons were partitioned into subnetworks and made preferential synaptic connections with members of the same subnetwork (Ko et al., 2011; Cossell et al., 2015; Muir and Mrsic-Flogel, 2015). These networks were otherwise identical to those shown in Figure 4. We compared the proportion of perturbed inhibitory neurons that were required to evoke a paradoxical effect between the uniform connectivity networks in Figure 4 and a network consisting of 10 subnetworks with selective connectivity (Fig. 5). Our results showed that the same or an even greater proportion of inhibitory neurons needed to be perturbed to evoke a paradoxical effect in networks with feature-specific connectivity (Fig. 5), especially when external input was not global, but rather was specific to one or several subnetworks.

### Perturbations in networks with spatial extent

Connections between cortical neurons have spatial extent and the density of connections between neurons is modulated by their relative locations within cortex (Hellwig, 2000; Holmgren et al., 2003; Boucsein et al., 2011; Levy and Reyes, 2012). Most experimental perturbations of neuronal activity are also spatially localized. Although a perturbation may target all inhibitory neurons in a particular location, it is possible that the spatial size of a perturbation determines whether a paradoxical inhibitory response occurs in an ISN. The physical size of a perturbation may then determine whether an ISN regime can be detected.

**Figure 6.**
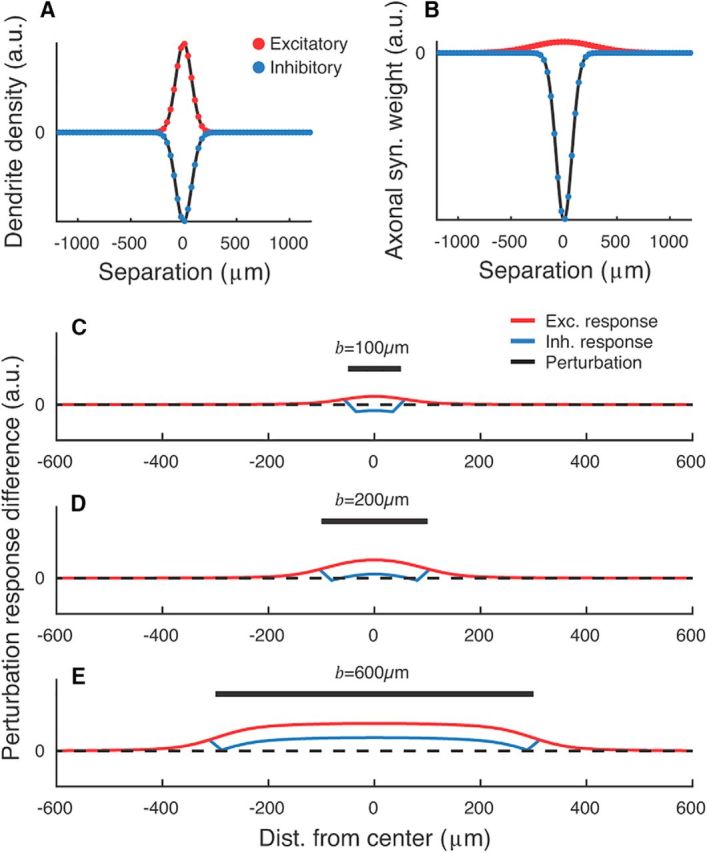
Physical size of an inhibitory perturbation determines whether a paradoxical effect will be displayed in networks with spatial extent. ***A***, ***B***, Cross-sections of 2D dendritic density fields (***A***) and axonal synaptic weight fields (***B***) for excitatory (red, positive) and inhibitory (blue, negative) neurons. Dots indicate the resolution of the simulation mesh. ***C***–***E***, Cross-section of spatial perturbations applied to neural fields in an ISN regime for varying perturbation diameter *b*. The inhibitory field was perturbed with δ = −0.2. For narrow perturbations, the perturbed inhibitory neurons do not show a paradoxical response even in the presence of an ISN regime (***C***; perturbation suppressed inhibitory activity). For wider perturbations, a paradoxical inhibitory response is evoked (***D***,***E***; perturbation increased inhibitory activity). ***C***–***E*** are shown on a common scale. Parameters are as in Table 1 and {*w_E_*, *w_I_*} = {4, 4}.

We simulated a neural field model with spatial extent and with neuronal connectivity modulated by Gaussian axonal and dendritic fields (Fig. 6*A*,*B*; see Materials and Methods). The model comprised two plates of neurons, one excitatory and one inhibitory, and implemented torus boundary conditions. We perturbed inhibitory neurons in circular regions of the model and measured the presence of a paradoxical effect in the center of the perturbation zone.

We found that, in networks operating in an ISN regime, narrow perturbations did not give rise to a paradoxical inhibitory response even in the center of the perturbation zone (Fig. 6*C*). Broader perturbations led to robust paradoxical responses in the center of the perturbation zone, with edge effects leading to a failure of the paradoxical inhibitory response at the limits of the perturbation (Fig. 6*D*,*E*). For a wide range of parameters, the minimum perturbation width needed to evoke a paradoxical inhibitory response was <250 μm.

### Inactivating the excitatory network may prevent detection of an ISN regime

For a network to be in an ISN regime, the excitatory network must be unstable in the absence of inhibition, which places a lower bound on the total synaptic output from individual excitatory neurons of *w_E_*(1 − *f*_I_) > 1. However, if only a portion of the excitatory network is active, then the effective excitatory synaptic drive available to the recurrent circuit will be lower than *w_E_*. This has two consequences for experimental perturbations: first, the excitatory network must be in an active state in order for an ISN regime to be detectable. Second, if an inhibitory perturbation leads to excitatory inactivation, this may complicate or prohibit the detection of an ISN regime.

**Figure 7.**
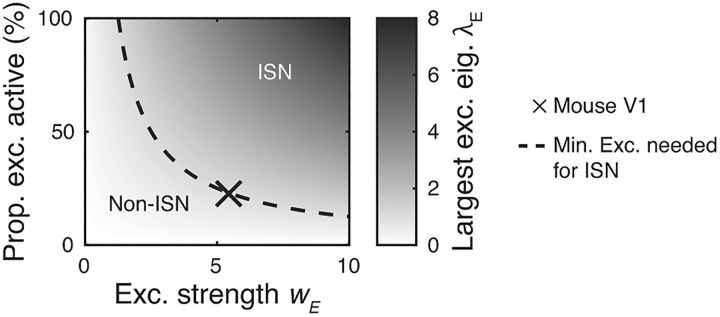
Depending on total excitatory strength *w_E_*, a minimum proportion of the excitatory population must be active for the network to operate in an ISN regime. For a given *w_E_*, the network will only operate in an ISN regime if the effective excitatory recurrence is strong enough to lead to excitatory instability (i.e., λ*_E_* > 1). Dashed line: λ*_E_* = 1. Parameters: *f_I_* = 20%. X: *w_E_* = 5.4 as in Figure 4. Prop., Proportion; exc., excitatory; eig., eigenvalue.

We computed the fraction of the excitatory network that must be active to place the network in an ISN regime for a given total excitatory strength *w_E_* (Fig. 7, dashed line). Under parameters estimated for mouse visual cortex (cross, *w_E_* = 5.4), at least 23% of the excitatory network must be active to permit the detection of an ISN regime. These results suggest that suppression rather than activation of inhibitory networks is likely to be the better strategy for revealing ISNs, particularity in the presence of the sparse activity states found in cortex.

### Measuring inhibitory input currents in excitatory neurons

Litwin-Kumar et al. (2016) proposed that recording the inhibitory current received by excitatory neurons as an experimentally accessible metric for observing the paradoxical effect of an ISN. Due to dense connectivity from the inhibitory population onto excitatory neurons (Fino and Yuste, 2011), recording net inhibitory currents provides an estimate of the mean activity of the local inhibitory population rather than sampling from an individual inhibitory neuron. Optogenetic perturbation of the inhibitory population while recording from individual excitatory neurons was performed by Atallah et al. (2012). However, the behavior of ISNs under simulated optogenetic perturbations is not known, leaving in question whether the averaging is sufficient in sparse networks and under what conditions a paradoxical effect should be visible.

**Figure 8.**
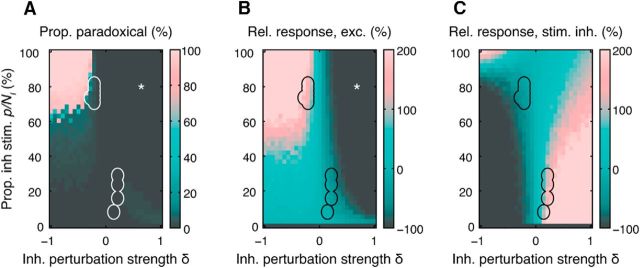
Paradoxical effects under simulated optogenetic perturbation. ***A***, Responses to perturbation in an ISN regime network indicating the proportion of excitatory neurons that exhibit a paradoxical effect in the net inhibitory input currents as a function of perturbation strength δ and proportion *p* of inhibitory neurons perturbed. ***B***, ***C***, Relative change in excitatory (***B***) and stimulated inhibitory (***C***) neuron activity for the same simulations as in ***A***. We considered that a paradoxical effect was visible when the input currents changed by at least 10% in the appropriate direction. Outlined regions in ***A***–***C*** indicate responses to perturbation where changes in excitatory and inhibitory activity are approximately equal to those reported by Atallah et al. (2012) (see Materials and Methods). *Region where the majority of excitatory neurons are below threshold, leading to failure of excitatory-driven inhibition. Parameters: {*w_E_*, *w_I_*, *h_EE_*, *h_EI_*, *h_IE_*, *h_II_*, *N_E_*, *N_I_*} = {4, 100, 6.4 × 10^−3^, 0.21, 0.24, 0.99, 4800, 1200}.

We therefore performed simulated optogenetic perturbations of the inhibitory population by injecting positive and negative currents and recording the resulting change in inhibitory input to excitatory neurons (Fig. 8). We simulated the presence of a stimulus in the network by providing random fixed input currents to each neuron. This placed the network in a realistic regime where symmetry is broken by an input stimulus and competition between neurons can be expressed. We then perturbed a randomly chosen proportion *p*/*N_I_* of the inhibitory network by providing a common input current with amplitude δ ranging (−1,1) designed to simulate perturbation by optogenetic activation or suppression.

We recorded the amplitude of inhibitory input currents impinging on each excitatory neuron and defined an excitatory neuron as showing a paradoxical effect if inhibitory input currents were modified by at least 10% in response to the inhibitory perturbation. As shown in Figure 8*A*, paradoxical effects were only observed in a substantial proportion of excitatory neurons when the majority of inhibitory neurons was inhibited. Indeed, regimes exist for ISN networks with strong excitatory and inhibitory feedback, where the paradoxical effect cannot be observed in the majority of excitatory neurons. Indicated regions in Figure 8 correspond to the effect sizes reported in Atallah et al. (2012), as determined by comparing the relative change in firing rates of excitatory and inhibitory neurons after a perturbation (Fig. 8*B*,*C*). Under a range of choices for strengths of excitation and inhibition, the simulated perturbations equivalent in size to those reported in Atallah et al. (2012) were not sufficient to demonstrate the paradoxical effect.

### Perturbations in networks with multiple inhibitory subclasses

Our results so far were obtained in network models including only a single inhibitory class. However, interneurons form multiple inhibitory subclasses in the neocortex (Pfeffer et al., 2013). Recently, Litwin-Kumar et al. (2016) examined the role of multiple inhibitory classes on network stability, with each class implemented as a single simulation node. They found that including additional inhibitory classes did not change the dynamics of inhibitory stabilization with regard to observing a paradoxical network response (their Figs. 1,2).

We therefore investigated how the dynamics of inhibitory perturbations changes in networks with an elaborated inhibitory system consisting of many neurons and separate inhibitory populations representing PV, SOM, and VIP inhibitory classes. We chose the parameters of connectivity similar to experimentally reported values (Pfeffer et al., 2013; Litwin-Kumar et al., 2016; see Materials and Methods). Because excitatory neurons became silent for strong connections between excitatory and SOM subpopulations, we varied this connectivity from weak to strong and evaluated the critical fraction of inhibition needed in each case to observe a paradoxical effect (for details, see Materials and Methods).

**Figure 9.**
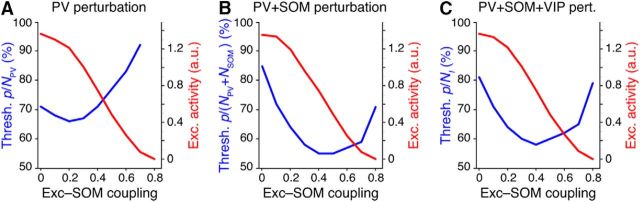
Fraction of inhibitory interneurons required to evoke a paradoxical effect in networks with multiple inhibitory subclasses. ***A***, Minimum proportion of PV subpopulation needed to be perturbed to evoke the paradoxical effect, as a function of the coupling strength of recurrent connections between excitatory cells (Exc.) and SOM neurons (see Materials and Methods; Eq. 7 in Litwin-Kumar et al., 2016). For very strong Exc.–SOM coupling values, excitatory activity (red) is completely silenced. No paradoxical effect can be observed in this state. For intermediate values of Exc.–SOM coupling, large fractions of PV neurons must be perturbed to evoke a paradoxical effect. ***B***, ***C***, Effect of simultaneously perturbing the PV and SOM (***B***) or PV, SOM, and VIP (***C***) subclasses. The same fraction of neurons was perturbed in each inhibitory class.

We found that perturbing a large fraction of PV neurons was also required to evoke a paradoxical effect in networks with multiple subclasses of inhibition (Fig. 9*A*). Moreover, perturbing the SOM and SOM+VIP subpopulations in addition to PV was more effective in evoking a paradoxical effect compared with perturbing PV alone (Fig. 9*B*,*C*). This was especially the case for intermediate coupling strength between excitatory and SOM populations (Exc.–SOM coupling ≈0.4). The critical fraction of inhibitory neurons that must be perturbed reduces to ≈55% for intermediate Exc.–SOM coupling strengths. For very strong Exc.–SOM coupling, excitatory activity was strongly suppressed, making inhibitory stabilization more difficult to detect.

These results confirm that that perturbation of a large fraction of inhibitory neurons is required to evoke a paradoxical effect in the realistic case of multiple inhibitory subclasses and where the PV inhibitory population comprises only a subset of inhibition in cortex. We therefore conclude that networks including multiple inhibitory classes behave in a qualitatively similar manner to those with a single inhibitory class.

### Perturbations in more realistic networks of spiking neurons

Our results so far were obtained in network models with simplified firing rate dynamics. However, networks composed of nonlinear spiking units are known to show rich and complex activity dynamics (Brunel, 2000; Ostojic, 2014), with response properties depending on the operating regime of activity (Destexhe et al., 2003; Kuhn et al., 2004; Kumar et al., 2008). To verify that our results hold in more biologically realistic networks, we investigated the dynamics of paradoxical inhibitory response in networks of nonlinear, conductance-based spiking neurons (see Materials and Methods).

**Figure 10.**
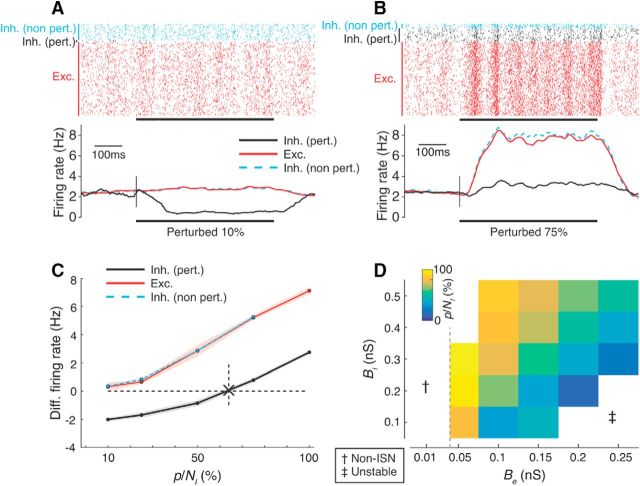
The paradoxical effect in spiking ISNs depends on the proportion of perturbed inhibitory neurons. ***A***, ***B***, Result of perturbing 10% (***A***) and 75% (***B***) of the inhibitory population in a spiking network model by reducing input to inhibitory neurons. Top, Single-trial spike rasters from the entire population. Bottom, Averaged firing rates over 10 trials (smoothed by a boxcar filter of 100 ms width). Black bar is the perturbation period (cf. Fig. 3). Red are excitatory (Exc.) neurons; black are perturbed inhibitory neurons (Inh. pert.); cyan are nonperturbed inhibitory neurons (Inh. non-pert.). Parameters for this network: {*N_E_*, *N_I_*, *B_e_*, *B_i_*} = {1600, 400, 0.1 nS, 0.2 nS}. For other parameters, see Materials and Methods and Table 3. ***C***, Mean (dots) and SD (shading) of the differential rates under a range of perturbed proportions for the network shown in ***A*** and ***B***. Cross and dashed line in ***C*** is the inferred minimum fraction of perturbed inhibition *p*/*N_I_* required to obtain the paradoxical effect (for details, see Materials and Methods). ***D***, Minimum fraction *p*/*N_I_* for spiking networks while varying *B_e_* and *B_i_* (cf. Fig. 4). Dashed line in ***D***: border of the ISN regime according to a simplified linear analysis of the network (see Materials and Methods). †Non-ISN regime; ‡unstable regime (firing rates >100 Hz).

The spiking activity of a sample network of conductance-based exponential integrate-and-fire neurons is shown in Figure 10*A* before and after perturbation of two different fractions of the inhibitory population. The perturbation was performed by decreasing input to a subset of inhibitory neurons.

The average activity within each subpopulation (excitatory, perturbed inhibitory, and unperturbed inhibitory) is shown in Figure 10*A* (bottom). When 10% of the inhibitory population was perturbed, no paradoxical effect was observed: the (negatively) perturbed inhibitory subpopulation decreased its activity, whereas the unperturbed inhibitory and excitatory subpopulations increased their activity. However, when larger fractions (75%) of inhibitory neurons were perturbed, the network displayed the paradoxical effect by increasing the average activity of the perturbed neurons despite a decrease in the input to the inhibitory network, consistent with the predictions of our firing rate model (cf. Figs. 10*B*, 4*A*).

To quantify the strength and presence of the paradoxical effect, we measured the average differential firing rate (perturbed rate minus baseline rate) while varying the fraction of perturbed inhibitory neurons (Fig. 10*C*; for details, see Materials and Methods). The paradoxical effect was present when large fractions of inhibitory neurons were perturbed, as indicated by a positive differential rate. We determined the minimum fraction at which the paradoxical effect emerged by interpolating the mean differential rate and inferring the point at which the differential rate crossed 0 (Fig. 10*D*; see Materials and Methods). Under these simulation conditions, >60% of the inhibitory neurons were required to generate a paradoxical effect.

We next investigated whether the minimum fraction of inhibitory neurons *p*/*N_I_* required to evoke the paradoxical effect depended on the relative strengths of excitatory and inhibitory feedback, as predicted by our nonspiking simulations. To test this, we fixed all parameters of the spiking network and modified the strength of excitatory and inhibitory conductances, *B_e_* and *B_i_*, respectively (Fig. 10*D*). For each combination of synaptic strengths, we estimated the minimum fraction of inhibition *p*/*N_I_* from the differential rate curves (analogous to Fig. 10*D*).

When excitation was too weak (Fig. 10*D*, left, white), no paradoxical effect was visible. For these values of excitation, the network was not operating in an ISN regime because the excitatory network alone was intrinsically stable (excitatory conductance *B*_e_ at and below gray vertical line obtained from the stability analysis of the linearized network; for details, see Materials and Methods). For very strong values of excitatory coupling without sufficient inhibitory feedback (high *B_e_* and low *B_i_*), networks underwent a transition from the stable regime with low firing rates and asynchronous, irregular activity to a regime with high firing rates and large pairwise correlations. This was consistent with our analysis of firing rate networks (cf. the unstable regime of network dynamics in Fig. 3). No paradoxical inhibitory response was observed in these unstable networks.

For intermediate values of *B_e_*, we found a smooth relationship between network parameters and the minimum fraction of perturbed inhibition *p*/*N_I_* required to see the paradoxical effect: networks with stronger excitation and weaker inhibition required smaller perturbations, similar to our results in firing rate networks (cf. Figs. 10*D*, 3). The trend for *p*/*N_I_* mimicked the tendency for the network to become unstable for strong *B_e_*. The results from our spiking simulations therefore agreed well with those from our analytical and firing rate models.

## Discussion

By examining the effects of simulated perturbations of activity in cortical network models with increasing degrees of realism, we determined what classes of perturbation could detect the computational regime of cortical networks successfully. In particular, we examined the properties of ISNs, which require inhibitory feedback to balance strong recurrent excitation (Tsodyks et al., 1997). This class of networks is particularly important for mammalian neocortex because many useful computational properties such as selective amplification, sharpening of tuning, and noise rejection require networks to be in an ISN regime (Douglas and Martin, 2007; Rutishauser and Douglas, 2009; Neftci et al., 2013; Muir and Cook, 2014; Hopfield, 2015).

In simple ISN models where each cell class is represented by a single unit, perturbation of the inhibitory unit reliably leads to a “paradoxical” inverse response whereby exciting an inhibitory neuron results in a net decrease in activity (Tsodyks et al., 1997; Litwin-Kumar et al., 2016; Fig. 2). We explored whether this paradoxical response could be used to detect ISNs experimentally by analyzing larger models with many neurons and with both homogeneous and sparse synaptic connectivity. We then tested the predictions arising from simplified firing rate models in more biologically realistic networks, including those with distance-dependent and subnetwork-specific connectivity, networks with multiple subtypes of inhibitory neurons, and conductance-based spiking network models. We found that, when inhibitory and excitatory populations are expanded, perturbing single inhibitory neurons only evokes a paradoxical response in very small networks.

In larger and more realistic networks, we found that eliciting a paradoxical inhibitory response requires a large fraction of the inhibitory population to be perturbed (Fig. 4). The proportion of cells required depends on the relative size and synaptic strengths of the excitatory and inhibitory populations but, importantly, not on the total size of the network. For networks with parameters estimated to be similar to mouse visual cortex, we found a large majority of inhibitory neurons must be perturbed to evoke a paradoxical response (>70%; Fig. 4*B*). Interestingly, connection sparsity does not affect the average minimum proportion of the inhibitory network that must be perturbed (Fig. 4*C*). Therefore, dense inhibitory feedback and sparse excitatory recurrence as present in mammalian cortex (Bock et al., 2011; Hofer et al., 2011; Martin, 2011; Bopp et al., 2014) does not imply that an ISN regime should be straightforward to observe. Our results suggest that establishing whether cortical networks operate in the ISN regime requires application of optogenetic strategies that allow perturbation of the vast majority of inhibitory interneurons in the circuit.

### Factors underlying the paradoxical effect in network models

Simplified network models (as in Tsodyks et al., 1997 and Litwin-Kumar et al., 2016) display robust paradoxical effects in response to perturbations of the inhibitory system. Because these networks use single neurons to represent the entire inhibitory population or entire inhibitory classes, they assume implicitly that global or class-global perturbations are made to the network. Our results demonstrate that this assumption is crucial to their results; we showed that networks operating in an ISN regime will not display a paradoxical inhibitory response unless a minimum proportion of the inhibitory population is perturbed (Fig. 4). Care is therefore needed in interpreting these earlier results in light of the complex inhibitory system in cortex.

We found that including sparsity in local recurrent connectivity did not change the minimum proportion of the inhibitory population that must be perturbed to evoke a paradoxical response (Fig. 4*C*). This is because the effects of sparse connectivity average out as the network size increases. Although the local minimum proportion of inhibitory neurons fluctuates across the network under sparse connectivity, we found that, if the average total excitatory and inhibitory synaptic strength per neuron is held fixed, then the average minimum proportion is then identical between fully and sparsely connected networks.

### Relationship to other balanced network models

Although instability of the excitatory subnetwork is not a required component of classical balanced networks (van Vreeswijk and Sompolinsky, 1996, 1998), they are usually assumed to operate in a regime where the net excitatory input to a single neuron in the absence of inhibition is well above its firing threshold; that is, a regime of unstable recurrent excitatory feedback. Because strong recurrent excitation is the most important determinant of a paradoxical effect in inhibitory stabilized networks, we therefore expect that our results hold in balanced networks with unstable excitation.

A more recent model is the stabilized supralinear network (SSN), which is an extension of classic ISNs to neuron models using nonlinear transfer functions (Ahmadian et al., 2013; Rubin et al., 2015). These networks can have multiple operating regimes depending on the average magnitude of input drive: if the network is only weakly driven, then its activity is determined by external input and weak recurrent interactions mediated by sublinear regions of the neuronal transfer function. Recurrent excitation is intrinsically stable in this mode, which implies the absence of an ISN regime and thus no paradoxical effect of inhibitory perturbation is expected. Conversely, if the external input is strong enough, then recurrent excitation is strengthened as a result of the nonlinear neuronal transfer function. Recurrent excitation is unstable in this regime, requiring inhibitory feedback for balance. In this regime, we expect SSNs to behave as we described in our results for ISNs.

### Application to experimental methods for inhibitory perturbation

#### Electrical stimulation

The activity of a neuron can be conveniently perturbed electrically by passing positive or negative currents through a recording electrode. However, because only small numbers of cells can be perturbed simultaneously using electrophysiological methods, our results suggest that paradoxical responses will not be observed in cortex using this method even if an ISN regime exists (Fig. 4).

#### Chemical stimulation

Several agonists and antagonists of GABA receptors exist, with varying selectivity for receptor subtypes (Chebib and Johnston, 1999; Krall et al., 2015). If such ant/agonists result in additive or subtractive modulation of inhibition, their effect is equivalent to adding or removing activity from both inhibitory and excitatory neurons. If the ant/agonists instead result in multiplicative or divisive modulation of inhibitory synaptic currents, the effect is equivalent to a modification of inhibitory weight. Our results for network global perturbations of input inhibitory currents or of inhibitory weight imply that ant/agonists with either mechanism of action cannot induce a paradoxical inhibitory response regardless of the presence of an ISN regime (Eq. 14,15).

#### Optogenetic perturbation

Optogenetic approaches enable photoactivation or photosuppression of specific neuron populations through genetically targeted expression of light-sensitive proteins (Boyden et al., 2005; Han and Boyden, 2007; Zhang et al., 2007; Aston-Jones and Deisseroth, 2013). This approach was taken by Atallah et al. (2012) to stimulate and suppress activity in PV-positive inhibitory neurons, coupled with simultaneous *in vivo* electrophysiology to record responses to stimulation in individual excitatory and inhibitory neurons. Atallah et al. (2012) showed that mild perturbation of PV neurons (∼−40% suppression and +20% activation; their Fig. 2) did not modify tuning of stimuli in mouse V1 (Atallah et al., 2012). The resulting changes in excitatory activity were also mild and inhibitory currents received by excitatory neurons did not show a paradoxical effect, on average (their Fig. 5).

Our findings cast new light on these results by showing that a large majority of inhibitory neurons must be perturbed to evoke a paradoxical response (Fig. 4). It is therefore not surprising that Atallah et al. (2012) did not observe such an effect, especially considering that PV inhibitory neurons comprise <50% of inhibitory neurons in the superficial layers of cortex (Markram et al., 2004; Gonchar et al., 2007) and a similar proportion of inhibitory synapses (Binzegger et al., 2004), placing a hard upper bound on the proportion of inhibitory neurons available for perturbation in their experiments.

We also showed that measuring inhibitory currents received by excitatory neurons (Litwin-Kumar et al., 2016) does not guarantee that a paradoxical effect will be observed in sparsely connected ISNs. In Figure 8, white outlines mark regimes of inhibitory perturbation that match the effects on excitatory and inhibitory activity observed by Atallah et al. (2012). In the presence of strong inhibition and sparse excitatory feedback in cortex, only a minority of excitatory neurons is expected to show a paradoxical effect in inhibitory input currents under this perturbation regime. The lack of a paradoxical change in inhibitory input currents observed by Atallah et al. (2012) therefore does not rule out the presence of an ISN regime in rodent cortex.

Our results suggest that optogenetic suppression of inhibitory neurons can be used to detect an ISN regime, but that optogenetic transducer proteins must be expressed in a majority of inhibitory neurons to do so. We found that suppression of inhibition is preferable to activation of inhibition if the goal is to detect an ISN regime (Figs. 7, 8). Activating inhibition leads to suppression of excitatory activity, reducing the effective recurrent excitatory drive in the network and preventing expression of ISN dynamics. We also found that the spatial size of a perturbation is expected to be important in ensuring a paradoxical inhibitory effect is evoked (Fig. 6), but that perturbations >250 μm in diameter are likely to evoke a robust paradoxical response.

We also found that, despite complex interactions between classes of inhibitory neurons in cortex, perturbing SOM and VIP neurons in addition to PV neurons was likely to lead to a more robust detection of an ISN regime. This could be achieved using multiple inhibitory class-specific promoters (e.g., PV-Cre × SOM-Cre × FLEXed virus) or a global inhibitory promoter such as glutamate decarboxylase to target all cells that synthesize GABA. Large area photostimulation could then be used to inhibit a large fraction of inhibitory neurons, rather than the subpopulation studied in Atallah et al. (2012) and the presence or absence of a paradoxical effect determined by examining inhibitory drive onto pyramidal cells (Litwin-Kumar et al., 2016). However, because our networks did not explore the effects of class-specific inhibition onto various subcellular compartments, potential network effects arising from differences between dendritic- and somatic-targeting inhibition must be weighed carefully.

Two recent studies inferred the presence of ISN regimes in the visual (Adesnik, 2017) and auditory (Kato et al., 2017) cortex of awake mice by observing an increase in synaptic inhibition to pyramidal cells as a result of optogenetically suppressing inhibitory neurons (SOM neurons in Adesnik, 2017 and SOM or PV neurons in Kato et al., 2017). This is generally consistent with the results of our model including multiple inhibitory subclasses (Fig. 9). However, an increase in inhibitory drive onto pyramidal cells could also be caused by disinhibition of PV neurons by inactivated SOM neurons (Adesnik, 2017). To safely infer the presence of an ISN regime from these results, future experimental and theoretical research is needed to rule out disinhibition.

#### Impact of anesthesia and external stimulation

Many anesthetics act to reduce overall neuronal excitability (Rudolph and Antkowiak, 2004) and effective connection strength (Campagna et al., 2003). In our models, this effectively leads to reducing both excitatory and inhibitory synaptic weights. In both cases, we expect networks to be more stable under anesthesia, with a weaker or absent expression of ISN properties such as the paradoxical response. Observing ISN properties is therefore likely to be easier in awake animals.

Care must also be taken to ensure an appropriate operating regime for cortex when probing for inhibitory stabilization. We found that, if the cortex is driven with an external stimulus biased in a similar way to local excitatory connection specificity, for example, a visual grating of a single orientation in rodent visual cortex, then detecting an ISN regime is more difficult. This result implies that inhibitory stabilization might be easier to detect under spontaneous activity or using stimuli that drive less structured cortical activity.

#### Other evidence for the operating regime of cortex

Surround suppression in cat visual cortex is consistent with an ISN operating regime under the assumption that projections from the visual surround specifically modulate the inhibitory population (Ozeki et al., 2009). Robust propagation of oscillatory activity in several species (Timofeev et al., 2000; Rubino et al., 2006; Wu et al., 2008; Stroh et al., 2013) suggests that recurrent excitation is strong enough to regenerate activity (Beurle, 1956; Compte et al., 2003; Wu et al., 2008). In the rodent, supralinear amplification of single spikes (London et al., 2010) provides additional evidence for strong excitatory recurrence in cortex. More directly, anatomical and physiological estimates of synaptic contributions from various neuronal classes place both cat and rodent cortex in an ISN regime (Binzegger et al., 2004; Binzegger et al., 2009; Lefort et al., 2009).

Our results illustrate that emergent dynamics in the highly recurrent networks of mammalian neocortex can complicate experimental detection of the network configuration. In particular, intuitions derived from reduced models about how classes of neurons interact may not hold in more realistic networks. Our analysis and simulation of larger scale models show that, although it is possible to test for an ISN regime in cortex using optogenetics, particular experimental conditions are required to do so successfully. Computational modeling of cortical dynamics is therefore an essential tool with which to predict the effect that perturbations will have under particular hypotheses of cortical interactions and to guide experimental design to test those hypotheses.
